# Diphtheria and Sewer Gas

**Published:** 1878-10

**Authors:** 


Diphtheria and Sewer-Gas.—Dr. W. Snively, physician to the Pittsburg (Pa.) Board of Health, lately read a paper on the cause of diphtheria in an outbreak of the disease in Pittsburg during the year 1877. Out of the 855 cases, the total number reported, there 366 deaths. Of these, 457, with 141 deaths, occurred in a particular district of the city in which the sewerage was almost criminally defective—*i. e.*, there was insufficient grade, inadequate diameter of the main at certain points, absence of traps at the street drops, private connection-points and main termini, while the manholes were tightly covered and there was an utter lack of ventilation. Slaughter-houses near the termini drained their offal and refuse through connections having no traps; and one of the latter emptied directly into an untrapped drop. Previous to last July, diphtheria had prevailed in only an ordinary number of cases. The epidemic set in during July, and raged about five months. During most of this period one main was choked for a distance exceeding 2,000 feet. Two heavy rainfalls occurred in July, two in August. Dr. S. infers from these data that sewer-gas emanations, propagating the disease in the poisoned human system, was the cause of that epidemic. He explains that heavy rains caused choking of the mains, and expulsion of stagnant sewer-gas into the dwellings through the private connection-pipes. He very properly insists that ventilation of the main is of the greatest importance in preventing accumulation of sewer-gas, and its consequences, no matter how well constructed the sewer may otherwise be. Undoubtedly thorough main ventilation is of primary importance.

But 'trapping' and ventilation of the *connection pipes* are of equal moment, when there is danger of heavy rains forcing the gas, which may have been generated, into the dwelling. Sewerage is a subject which has been sadly neglected in practice, but we hope that later day experiences, and the valuable advice of our sanitary authorities, may soon bring forth ripe fruit and thus add another triumph to those of preventive medicine.—*The Medical Record.*

